# Socio-demographic characteristics associated with emotional and social loneliness among older adults

**DOI:** 10.1186/s12877-021-02058-4

**Published:** 2021-02-09

**Authors:** Irene N. Fierloos, Siok Swan Tan, Greg Williams, Tamara Alhambra-Borrás, Elin Koppelaar, Lovorka Bilajac, Arpana Verma, Athina Markaki, Francesco Mattace-Raso, Vanja Vasiljev, Carmen B. Franse, Hein Raat

**Affiliations:** 1 Department of Public Health, Erasmus University Medical Center, P.O. Box 2040, 3000 CA Rotterdam, The Netherlands; 2 Manchester Urban Collaboration on Health, Centre for Epidemiology, Division of Population Health, Health Services Research and Primary Care, Manchester Academic Health Science Centre, The University of Manchester, Manchester, UK; 3 Polibienestar Research Institute, University of Valencia, Valencia, Spain; 4 Research Centre Innovation in Care, Rotterdam University of Applied Sciences Rotterdam, Rotterdam, The Netherlands; 5 Department of Social Medicine and Epidemiology, Faculty of Medicine, University of Rijeka, Rijeka, Croatia; 6 Alliance for Integrated Care, Athens, Greece; 7 Division of Geriatrics, Department of Internal Medicine, Erasmus MC University Medical Center, Rotterdam, The Netherlands

**Keywords:** Emotional loneliness, Social loneliness, Population characteristics

## Abstract

**Background:**

International studies provide an overview of socio-demographic characteristics associated with loneliness among older adults, but few studies distinguished between emotional and social loneliness. This study examined socio-demographic characteristics associated with emotional and social loneliness.

**Methods:**

Data of 2251 community-dwelling older adults, included at the baseline measure of the Urban Health Centers Europe (UHCE) project, were analysed. Loneliness was measured with the 6-item De Jong-Gierveld Loneliness Scale. Multivariable logistic regression models were used to evaluate associations between age, sex, living situation, educational level, migration background, and loneliness.

**Results:**

The mean age of participants was 79.7 years (SD = 5.6 years); 60.4% women. Emotional and social loneliness were reported by 29.2 and 26.7% of the participants; 13.6% experienced emotional and social loneliness simultaneously. Older age (OR: 1.16, 95% CI: 1.06–1.28), living without a partner (2.16, 95% CI: 1.73–2.70), and having a low educational level (OR: 1.82, 95% CI: 1.21–2.73), were associated with increased emotional loneliness. Women living with a partner were more prone to emotional loneliness than men living with a partner (OR: 1.78, 95% CI: 1.31–2.40). Older age (OR: 1.11, 95% CI: 1.00–1.22) and having a low educational level (OR: 1.77, 95% CI: 1.14–2.74) were associated with increased social loneliness. Men living without a partner were more prone to social loneliness than men living with a partner (OR: 1.94, 95% CI: 1.35–2.78).

**Conclusions:**

Socio-demographic characteristics associated with emotional and social loneliness differed regarding sex and living situation. Researchers, policy makers, and healthcare professionals should be aware that emotional and social loneliness may affect older adults with different socio-demographic characteristics.

**Supplementary Information:**

The online version contains supplementary material available at 10.1186/s12877-021-02058-4.

## Background

Loneliness can be defined as an unpleasant experience, occurring when the quantity or quality of a person’s social relationships is perceived to be deficient [1]. In general, feelings of loneliness motivate people to strengthen their existing social relationships or to build new relationships, after which these negative feelings may diminish [2]. However, for some people loneliness can become a chronic state. Persistent loneliness has been associated with negative outcomes for mental and physical health, such as depression, psychological distress, reduced self-esteem, cognitive impairment, functional decline, high blood pressure, cardiovascular diseases, and higher mortality rates [2–7].

Based on data collected in the third round (2006–07) of the European Social Survey, Yang and Victor [8] found that the prevalence of frequent loneliness among citizens aged 60 years and older, defined as feeling lonely ‘most of the time’ or ‘all or almost all the time’, varied between 19 and 34% in Eastern Europe, 10–15% in Southern Europe, and 3–9% in Northern Europe. The prevalence of frequent loneliness was highest among adults aged 80 years and older [8]. Age-related changes and losses, such as deteriorating health, declining mobility, changing social roles, and the loss of a partner or friends have been associated with an increased susceptibility of loneliness in older age [4]. As European populations are ageing [9], loneliness can be expected to be a growing public health issue.

International studies provide an overview of socio-demographic characteristics associated with increased overall loneliness among older adults, such as widowhood, living in disadvantaged socioeconomic circumstances and having a migration background [4, 10–13].

However, few studies have distinguished between different dimensions of loneliness, such as emotional and social loneliness [3]. In 1973, Weiss [14] proposed that emotional loneliness is related to an absence of intimate attachments to other persons, whereas social loneliness is related to an absence of an engaging social network or a lack of social integration [14]. The onset of emotional loneliness may be related to the loss of intimate relationships, for example by a divorce or widowhood [14]. The onset of social loneliness may be related to the loss of a network of social relationships, for example by moving to another place [14]. Weiss [14] suggested that emotional loneliness may be characterized by feelings of isolation and anxiety, while social loneliness may be characterized by feelings of boredom, aimlessness, and marginality. Previous studies indicate that, despite being correlated, emotional and social loneliness can be recognized as distinct states affecting different groups of people [15–22].

The distinction between emotional and social loneliness may be relevant for the development of intervention strategies to reduce loneliness. According to the theoretical framework of Weiss, emotional loneliness may only be alleviated by a new or recovered intimate relationship, providing a sense of attachment, and social loneliness may only be alleviated by (re-)entering a social network, providing a sense of social integration [14, 21]. Many studies on the effects of intervention strategies did not report the impact on emotional and social loneliness [23]. In their meta-analysis, Masi, Chen [23] found that interventions to increase opportunities for social interaction or enhance social support had relatively small effects on reducing overall loneliness. Perissinotto, Holt-Lunstad [24] suggested that many interventions to reduce loneliness focus on the establishment of new social contacts, while this may only be beneficial for people who experience loneliness due to a lack of social contacts [24]. Interventions taking into account factors associated with the onset of loneliness, may be more appropriate [23]. Bouwman and Van Tilburg [25] have distinguished four intervention goals: 1) having a social network (related to social loneliness), 2) experiencing a sense of belonging (related to social loneliness), 3) experiencing meaning (related to emotional loneliness), and 4) experiencing intimacy (related to emotional loneliness). Intervention strategies may be used to reach multiple or specific intervention goals. For example, social skills training might be used to improve a person’s social network; befriending interventions might be used to increase a sense of belonging; a voluntary job might be used to increase a sense of meaning [25].

In the current study, we examine which groups of older adults are at risk of emotional and social loneliness. We make a distinction between emotional and social loneliness because each dimension may require specific intervention strategies. Our study provides insight into the potential target groups for intervention strategies addressing emotional and/ or social dimensions of loneliness. The following research question is answered: Which socio-demographic characteristics are associated with emotional and social loneliness among older adults?

## Methods

### Data

Participants were 2325 community-dwelling older adults included in the baseline measure of the Urban Health Centres Europe (UHCE) project, a pre-post controlled trial to evaluate coordinated preventive care aiming to promote healthy ageing [26]. Multiple integrated care pathways (interventions) were implemented in the urban areas of Greater Manchester, The United Kingdom; Pallini, Greece; Rijeka, Croatia; Rotterdam, The Netherlands; Valencia, Spain. The UHCE study was approved by Medical Ethics Committees in all participating countries. UHCE was registered as ISRCTN52788952 [26]. Participants were recruited between May 2015 and June 2017. Eligible participants were (1) aged 75 years or older (≥70 years in Greece and Spain), (2) living independently in selected neighbourhoods, (3) able to understand the local language, and (4) able to cognitively evaluate the risks and benefits of participation. All participants provided written informed consent. A full description of the inclusion process has been provided elsewhere [27]. The current study used baseline data collected by self-reported questionnaires. Participants with missing data of the De Jong-Gierveld Loneliness Scale (*n* = 35) or of socio-demographic characteristics (*n* = 39), were excluded. Hence, the population for analyses consisted of 2251 participants. Loneliness was measured by the 6-item De Jong-Gierveld Loneliness Scale [28] (Additional Table 1), a valid and reliable instrument to assess emotional and social loneliness among older adults living in diverse countries [29]. Response categories were ‘no’, ‘more or less’ and ‘yes’. Scores were calculated according to the guidelines [28]. Scores varied between 0 ‘No loneliness’ and 3 ‘Intense emotional or social loneliness’Scores ≥2 indicating moderately intense or intense emotional loneliness were categorized as emotional and social loneliness [28]. The Loneliness Scale was originally developed in Dutch [30]. Validated translations were available in English and in Spanish. Forward-backward translation procedures were applied to translate the items from English into Croatian and Greek; the items were pilot tested among local older citizens. We used Cronbach’s α to evaluate internal consistency of the 6-item scale and 3-item sub scales for each country (Additional Table 2). The Cronbach’s α for the 6-item scale ranged between .62 (The United Kingdom) and .78 (The Netherlands). The Cronbach’s α for the 3-item sub scale for emotional loneliness ranged between .56 (The United Kingdom) and .75 (The Netherlands). The Cronbach’s α for the 3-item sub scale for social loneliness ranged between .68 (The United Kingdom) and .81 (Greece).

The following socio-demographic characteristics were studied: age (in years), sex (male/female), living situation (living with a partner/ living without a partner), educational level, and migration background. Educational level was divided into three categories, based on the International Standard Classification of Education (ISCED). ISCED level 0–1 was categorized as ‘primary or lower’; ISCED level 2–5 was categorized as ‘secondary’; ISCED level 6–8 was categorized as ‘tertiary’ [31]. Migration background was assessed by country of birth. A country of birth other than the country of residence was categorized as a migration background. The country of residence was included as a covariate.

### Statistical analysis

Descriptive statistics were used to characterize the participants. Multivariable logistic regression models were used to assess independent associations between socio-demographic characteristics and emotional, respectively, social loneliness. Odds ratios (ORs) and 95% confidence intervals (95% CI) were calculated for each factor. To assess moderating effects of sex, living situation and country for each factor, interaction terms were separately added to the multivariable models on emotional and social loneliness. Significant interaction effects were presented according to guidelines of Knol and VanderWeele [32].

A sensitivity analysis was conducted to examine whether associations between socio-demographic characteristics and loneliness differed when using a broader (cut-off value ≥1) and a stricter definition (cut-off value =3) of loneliness (Additional Table 3). Data were analysed in Statistical Package for Social Sciences, version 25 for Windows (IBM SPSS Statistics for Windows, IBM Corp). *P*-values below .05 were considered to be statistically significant.

### Nonresponse analysis

Characteristics of participants with missing data (*n* = 74) were compared with characteristics of participants with complete data (*n* = 2251) using chi-squared tests. Participants with missing data more often had a migration background (*P* < .001), and more often lived with a partner (*P* = .010) than participants without missing data. There were no differences regarding other socio-demographic characteristics.

## Results

### Characteristics of the participants

The socio-demographic characteristics of the participants are presented in Table 1. The mean age of participants was 79.7 years (SD = 5.6 years), 60.4% of the participants were women and 50.4% lived with a partner.

**Table 1:** Socio-demographic characteristics of 2251 participants of the UHCE study; by emotional and social loneliness

Socio-demographiccharacteristics	Total	Emotional loneliness (yes)	Emotional loneliness (no)		Social loneliness (yes)	Social loneliness (no)	
	*n* = 2251 (100%)	*n* = 657 (29.2%)	*n* = 1594 (70.8%)	*P*-value	*n* = 600 (26.7%)	*n* = 1651 (73.3%)	*P*-value
	Mean (SD) N (% of total)	Mean (SD) N (% ‘lonely’)	Mean (SD) N (% ‘not lonely’)		Mean (SD) N (% ‘lonely’)	Mean (SD) N (% ‘not lonely’)	
*Age (in years)*	79.7 (SD = 5.6)	80.2 (SD = 5.8)	79.5 (SD = 5.6)	**.006**	80.5 (SD = 5.3)	79.4 (SD = 5.8)	**<.001**
*Sex*				**<.001**			.223
Female	1360 (60.4%)	463 (34.0%)	897 (66.0%)		375 (27.6%)	985 (72.4%)	
Male	891 (39.6%)	194 (21.8%)	697 (78.2%)		225 (25.3%)	666 (74.7%)	
*Living situation*				**<.001**			**.002**
With partner	1135 (50.4%)	238 (21.0%)	897 (79.0%)		270 (23.8%)	865 (76.2%)	
Without partner	1116 (49.6%)	419 (37.5%)	697 (62.5%)		330 (29.6%)	786 (70.4%)	
*Educational level*				**<.001**			**.006**
Tertiary	206 (9.2%)	43 (20.9%)	163 (79.1%)		41 (19.9%)	165 (80.1%)	
Secondary	1426 (63.3%)	397 (27.8%)	1029 (72.2%)		410 (28.8%)	1016 (71.2%)	
Primary or less	619 (27.5%)	217 (35.1%)	402 (64.9%)		149 (24.1%)	470 (75.9%)	
*Migration background*				.499			**.002**
No	2066 (91.8%)	599 (29.0%)	1467 (71.0%)		533 (25.8%)	1533 (74.2%)	
Yes	185 (8.2%)	58 (31.4%)	127 (68.6%)		67 (36.2%)	118 (63.8%)	
*Country*				**<.001**			**<.001**
The UK	548 (24.3%)	101 (18.4%)	447 (81.6%)		75 (13.7%)	473 (86.3%)	
Greece	345 (15.3%)	146 (42.3%)	199 (57.7%)		77 (22.3%)	268 (77.7%)	
Croatia	495 (22.0%)	202 (40.8%)	293 (59.2%)		278 (56.2%)	217 (43.8%)	
The Netherlands	369 (16.4%)	95 (25.7%)	274 (74.3%)		79 (21.4%)	290 (78.6%)	
Spain	494 (21.9%)	113 (22.9%)	381 (77.1%)		91 (18.4%)	403 (81.6%)	

*P*-values <.05 in bold. *P*-values for continuous variables were calculated with ANOVA and *P*-values for categorical variables were calculated with Chi-squared tests. *SD* standard deviation

Emotional loneliness was reported by 29.2% (*n* = 657) of the participants and social loneliness by 26.7% (*n* = 600) of the participants; 13.6% (*n* = 306) experienced emotional and social loneliness simultaneously, 15.6% (*n* = 351) experienced emotional loneliness exclusively, and 13.1% (*n* = 303) experienced social loneliness exclusively; 57.8% (*n* = 1300) reported neither emotional or social loneliness. Women (*P* < .001), participants living without a partner (*P* < .001), and participants with a lower educational level (*P* < .001) more often reported emotional loneliness. Participants living without a partner (*P* = .002), participants with a lower educational level (*P* = .006), and participants with a migration background (*P* = .002) more often reported social loneliness.

### Associations between socio-demographic characteristics and emotional loneliness

The multivariable model for emotional loneliness is presented in Table 2. Results of the interaction analysis for emotional loneliness are presented in Table 3. Significant interaction effects were found between sex and living situation (*P* = .013), and between country and living situation (*P* < .001). There were no other interaction effects for emotional loneliness (*p* > .05). The multivariable model for emotional loneliness showed that older age (OR per 5 years: 1.16, 95% CI: 1.06–1.28) was associated with increased emotional loneliness. Older adults with a primary or lower educational level had 1.82 (95% CI: 1.21–2.73) times higher odds of experiencing emotional loneliness compared to older adults with a tertiary educational level. Having a migration background was not associated with emotional loneliness.

**Table 2:** Multivariable logistic regression models on associations between socio-demographic characteristics and emotional and social loneliness among 2251 participants of the UHCE study

	Emotional loneliness (‘yes’ *n* = 657; 29.2%)	Social loneliness (‘yes’ *n* = 600; 26.7%)
	Multivariable model OR (95% CI)	Multivariable model OR (95% CI)
*Age (per 5 years)*	**1.16 (1.06–1.28)**	1.11 (1.00–1.22)
*Sex (female)*	**1.38 (1.10–1.72)**	0.86 (0.69–1.09)
*Living situation (without partner)*	**2.16 (1.73–2.70)**	1.23 (0.98–1.55)
*Educational level*		
Tertiary	ref.	ref.
Secondary	1.22 (0.83–1.79)	1.31 (0.88–1.95)
Primary or lower	**1.82 (1.21–2.73)**	**1.77 (1.14–2.74)**
*Migration background (yes)*	0.99 (0.69–1.41)	1.03 (0.72–1.48)
*Country*		
The UK	ref.	ref.
Greece	**4.33 (2.96–6.34)**	**1.88 (1.24–2.85)**
Croatia	**3.11 (2.32–4.16)**	**8.34 (6.14–11.33)**
The Netherlands	**1.50 (1.07–2.09)**	**1.66 (1.16–2.37)**
Spain	1.26 (0.86–1.84)	1.34 (0.89–2.00)

Odds ratios and 95% confidence intervals are derived from multivariable logistic regression analyses for emotional and social loneliness (cut-off value Loneliness sub scales ≥2). *P*-values <.05 in bold. *OR* odds ratio, *CI* confidence interval, *ref.* reference group

**Table 3:** *P*-values of the interaction terms added to the multivariable logistic regression models on associations between socio-demographic characteristics and emotional and social loneliness among 2251 participants of the UHCE study

Interaction terms	Emotional loneliness	Social loneliness
	*P*-value	*P*-value
Sex*age	.362	.482
Sex*country	.083	.684
Sex*living situation	**.013**	**.002**
Sex*educational level	.256	.298
Sex*migration background	.124	.272
Living situation*age	.562	.693
Living situation*country	**<.001**	.693
Living situation*educational level	.250	.841
Living situation*migration background	.558	.171
Country*age	.322	.854
Country*educational level	.058	.290
Country*migration background	.907	.620

*P*-values were derived by separately adding the interaction terms to the multivariable logistic regression models for emotional and social loneliness (presented in Table 2). *P*-values <.05 in bold

Table 4 presents the odds ratios for emotional loneliness by sex and living situation. Women living with a partner had 1.78 (95% CI: 1.31–2.40) times higher odds of experiencing emotional loneliness compared to men living with a partner. Living without a partner was associated with increased emotional loneliness among men and women. Men living without a partner had 3.10 (95% CI: 2.17–4.40) times higher odds of experiencing emotional loneliness compared to men living with a partner. Women living without a partner had 1.79 (95% CI: 1.37–2.33) times higher odds of experiencing emotional loneliness compared to women living with a partner.

**Table 4:** Results of analysis on interaction between sex and living situation for emotional loneliness among 2251 participants of the UHCE study

Emotional loneliness					
	*Living with partner*		*Living without partner*		OR (95% CI) for living without partner vs living with partner within strata of sex
	N lonely/not lonely	OR (95% CI)	N lonely/not lonely	OR (95% CI)	
Male	113/546	ref.	81/151	**3.10 (2.17–4.40)**	**3.10 (2.17–4.40)**
Female	125/351	**1.78 (1.31–2.40)**	338/546	**3.17 (2.45–4.12)**	**1.79 (1.37–2.33)**
**OR (95% CI) for female vs male within strata of living situation**		**1.78 (1.31–2.40)**		1.02 (0.74–1.41)	

Interaction between sex and living situation on the multiplicative scale *P* = .013. ORs were presented for each stratum with male living with a partner as the reference group. ORs for sex were presented within strata of living situation; ORs for living situation were presented within strata of sex. ORs are adjusted for age, educational level, migration background and country. *P*-values <.05 in bold. *OR* odds ratio, *CI* confidence interval, *ref*. reference group

Table 5 presents the odds ratios for emotional loneliness by country and living situation. In The United Kingdom (OR: 3.19, 95% CI: 1.85–5.47), Greece (OR: 2.45, 95% CI: 1.51–3.98), The Netherlands (OR: 5.31, 95% CI: 3.00–9.40), and Spain (OR: 1.95, 95% CI: 1.25–3.02), living without a partner was associated with increased emotional loneliness. In Croatia, living without a partner was not associated with increased emotional loneliness (OR: 1.10; 95% CI: .75–1.61).

**Table 5:** Results of analysis on interaction between country and living situation for emotional loneliness among 2251 participants of the UHCE study

Emotional loneliness					
	*Living with partner*		*Living without partner*		OR (95% CI) for living without partner vs living with partner within strata of country
	*N* lonely/not lonely	OR (95% CI)	*N* lonely/not lonely	OR (95% CI)	
The UK	19/210	ref.	82/237	**3.19 (1.85–5.47)**	**3.19 (1.85–5.47)**
Greece	81/157	**5.53 (3.10–9.84)**	65/42	**13.56 (7.11–25.87)**	**2.45 (1.51–3.98)**
Croatia	75/130	**6.31 (3.63–10.95)**	127/163	**6.93 (4.05–11.85)**	1.10 (0.75–1.61)
The Netherlands	19/171	1.11 (0.56–2.17)	76/103	**5.87 (3.31–10.41)**	**5.31 (3.00–9.40)**
Spain	44/229	1.78 (0.97–3.28)	69/152	**3.46 (1.91–6.28)**	**1.95 (1.25–3.02)**

Interaction between country and living situation on the multiplicative scale *P* < .001

ORs are adjusted for age, sex, educational level and migration background. *P*-values <.05 in bold. *OR* odds ratio, *CI* confidence interval, *ref.* reference group

### Associations between socio-demographic characteristics and social loneliness

The multivariable model for social loneliness is presented in Table 2. Results of the interaction analysis for social loneliness are presented in Table 3. A significant interaction effect was found between sex and living situation (*P* = .002). There were no other interaction effects for social loneliness (*p* > .05).

The multivariable model for social loneliness showed older age was associated with increased social loneliness (OR per 5 years: 1.11, 95% CI: 1.00–1.22), but non-significant (*P* = .057). Older adults with a primary or lower educational level had 1.77 (95% CI: 1.14–2.74) times higher odds of experiencing social loneliness compared to older adults with a tertiary educational level. Having a migration background was not associated with social loneliness.

Table 6 presents the odds ratios for social loneliness by sex and living situation. Men living without a partner had 1.94 (95% CI: 1.35–2.78) times higher odds of experiencing social loneliness compared to men living with a partner. Among women, living without a partner was not associated with increased social loneliness.

**Table 6:** Results of analysis on interaction between sex and living situation for social loneliness among 2251 participants of the UHCE study

Social loneliness					
	*Living with partner*		*Living without partner*		OR (95% CI) for living without partner vs living with partner within strata of sex
	*N* lonely/not lonely	OR (95% CI)	*N* lonely/not lonely	OR (95% CI)	
Male	148/511	ref.	77/155	**1.94 (1.35–2.78)**	**1.94 (1.35–2.78)**
Female	122/354	1.18 (0.87–1.59)	253/631	1.11 (0.86–1.43)	0.94 (0.71–1.25)
**OR (95% CI) for female vs male within strata of living situation**		1.18 (0.87–1.59)		**0.57 (0.41–0.80)**	

Interaction between sex and living situation on the multiplicative scale *P* = .002

ORs are adjusted for age, educational level, migration background and country. *P*-values <.05 in bold. *OR* odds ratio, *CI* confidence interval, *ref.* reference group

### Simultaneous emotional and social loneliness

In the study population, some participants only reported the presence of one type of loneliness, while others reported the presence of both types of loneliness. To explore whether the associations were different when only one type of loneliness was reported, or when both types of loneliness were reported, we performed additional analyses in which we compared these groups with older adults experiencing neither emotional or social loneliness (Additional Tables 4 and 5). The directions of the associations for exclusive emotional loneliness and exclusive social loneliness were similar. For the simultaneous experience of emotional and social loneliness, the results were similar to the results of emotional loneliness.

### Sensitivity analysis

The multivariable models using a broader and a stricter definition of loneliness are presented in Additional Table 3. The directions of the associations between socio-demographic characteristics and emotional and social loneliness were similar when using ≥1, ≥2, and = 3 as cut-off scores for the Loneliness sub scales.

## Discussion and conclusions

Older age, living without a partner, and having a low educational level were independently associated with increased emotional loneliness among older adults. Women living with a partner were more prone to emotional loneliness than men living with a partner. Older age and having a low educational level were associated with increased social loneliness. Men living without a partner were more prone to social loneliness than men living with a partner. With regard to the simultaneous experience of emotional and social loneliness, the results were similar to the results of emotional loneliness.

In our study among participants with a mean age of almost 80 years, older age was associated with increased emotional loneliness and with increased social loneliness (borderline significance). Results of a meta-analysis [10] showed that older age was associated with increased overall loneliness in studies among participants with a mean age > 80 years, but not among participants with a mean age of 60–80 years. Further research could examine possible differences in age-related factors associated with emotional and social loneliness, and their onset. The death of a partner may primarily be associated with emotional loneliness, whereas leaving paid employment and decreasing out-door mobility could be age-related factors primarily associated with social loneliness [33].

Women living with a partner were more prone to emotional loneliness than men living with a partner. This is in line with results of previous studies [34, 35]. Dykstra and de Jong Gierveld [35] have suggested that married men might more often rely on their partner for emotional support than married women, and tend to experience more emotional fulfilment in marriage [34, 35]. Distinguishing between emotional and social loneliness in future studies, and testing for interactions between sex and living situation or marital status, may clarify the association between sex and loneliness [10].

Living without a partner was associated with increased emotional loneliness, which is in line with results of previous studies [15, 18]. In addition, living without a partner was associated with increased social loneliness among men. This corresponds to findings of Dykstra and Fokkema [34], as results of their study showed that divorced men had a greater vulnerability to social loneliness than divorced women. Dykstra and Fokkema [34] have suggested that married women might more often be in charge of social activities, have bigger and more varied social networks and are less likely to lose social contacts after a divorce [34, 35].

In Croatia, living without a partner was not associated with increased emotional loneliness. The association between living situation and loneliness may be influenced by cultural norms and values, affecting individual expectations of family members [36]. Further research is needed to explain cross-country differences in the association between living situation and emotional loneliness. Descriptive statistics on emotional loneliness distinguishing between participants living with/ without children have been provided in Additional Table 6.

In general, data used in this study indicate that the proportion of older persons experiencing exclusive or combined emotional and social loneliness varies between European countries (Additional Table 4). Hansen and Slagsvold [35] suggest cross-country differences in the risk of late-life loneliness can be explained by macro-level inequalities in health, socioeconomic status, marital status, and social integration. In addition, cross-country differences may be explained by differences in social welfare, demographic composition, and cultural norms and values [36]. Having a low educational level was associated with increased emotional and social loneliness, which is in line with results of previous studies [4, 10, 15, 37]. Older adults with a low educational level are more likely to live in disadvantaged socioeconomic circumstances, which have been associated with chronic stress and a decreased quality of social relations [38]. In addition, living in disadvantaged socioeconomic circumstances has been associated with reduced opportunities for participation in social activities [4].

Having a migration background was not associated with loneliness, in contrast to results of previous studies [39, 40]. Older adults with a migration background might be at increased risk of loneliness as a result of language barriers, cultural differences, possible encounters with discrimination and racism, and a dislocation of social networks after transnational migration [39, 40]. In our study, social loneliness was reported more frequently by participants with a migration background, but there was no independent association. This may have differed between immigrant groups, depending on their command of the local language and the magnitude of cultural differences [39]. However, in our study the number of participants with a migration background was too low to distinguish between groups.

### Methodological considerations

A strength of this study was the relatively high average age of the study population. Older adults living in urban areas in Southern, Western and Eastern European countries were represented in the sample, which has increased the external validity of the results. Using a broader and a stricter definition of loneliness yielded similar results, indicating that the findings are applicable to older adults experiencing loneliness at different intensities.

Nevertheless, several limitations need to be considered when interpreting the findings. First, a sampling bias cannot be ruled out. Older adults with poor health may have been less likely to participate in the UHCE study [27], participants living with a partner and participants with a migration background were more often excluded from the population for analyses due to missing data. This has reduced the representativeness of the sample, and should be considered when the findings are generalized. Secondly, although previous studies reported good psychometric properties of the 6-item De Jong-Gierveld Loneliness Scale among culturally diverse groups, in our study, the internal consistency of the emotional loneliness sub scale was relatively low in the United Kingdom and Greece. We therefore recommend to consider the use of the 11-item De Jong Gierveld Loneliness scale in future studies distinguishing between emotional and social loneliness [28, 30].

Thirdly, the number of participants with a migration background was relatively low, and several sub groups in the interaction analyses were small, which may have resulted in a lack of statistical power to evaluate differences. In the multivariable logistic regression models, some 95% confidence intervals were relatively large. Future studies should expand upon the findings using longitudinal designs with large and varied samples of older adults. Lastly, causal directions of the associations between socio-demographic characteristics and loneliness could not be examined. Longitudinal research is needed to evaluate (bi-)directional associations between living situation, educational level, migration background and loneliness.

### Implications for policy and practice

Our findings may be used to identify target groups for intervention strategies aimed at the emotional or social dimension of loneliness. As mentioned above, Bouwman and Van Tilburg [25] have distinguished four intervention goals. Based on the results of this study, we hypothesize that intervention strategies aiming 1) to build or to strengthen a social network, and intervention strategies aiming 2) to increase a sense of belonging could in particular be beneficial for older adults, for men living without a partner, and for older adults with a low educational level. Intervention strategies aiming 3) to increase a sense of meaning, and intervention strategies aiming 4) to increase intimacy in relationships could in particular be beneficial for older adults of higher age, for older adults living without a partner, for women living with a partner, and for older adults with a low educational level. Future studies need to evaluate which intervention strategies are most effective in reducing emotional and/ or social loneliness [22].

## Conclusions

Older adults of higher age, women living with a partner, older adults living without a partner, and older adults with a low educational level may be at increased risk of emotional loneliness. Older adults of higher age, men living without a partner, and older adults with a low educational level may be at increased risk of social loneliness. More research in diverse populations, using longitudinal designs, is needed to confirm these findings. In the meantime, healthcare professionals and policy makers are advised to pay attention to an increased susceptibility of emotional and social loneliness in the above mentioned sub groups. We recommend to further develop effective and feasible interventions to prevent and alleviate specific dimensions of loneliness among older adults to contribute to their health and wellbeing.

## Supplementary Information

**Additional file1: Supplementary tables** Socio-demographic factors associated with emotional and social loneliness among older adults.

## Data Availability

The datasets used and/or analysed during the current study are available from the corresponding author on reasonable request.

## Abbreviations

UHCE: Urban Health Centers Europe
ISCED: International Standard Classification of Education
OR: Odds ratio
B: Beta
95% CI: 95% confidence interval
α: Alpha
