# Supplementary material for: Socio-demographic characteristics associated with emotional and social loneliness among older adults
Source: BMC Geriatr. 2021 Feb 9;21:114. doi: 10.1186/s12877-021-02058-4 (PMC7871533; doi:10.1186/s12877-021-02058-4)
Supplement: Supplementary file 1 — Additional file1: Supplementary tables Socio-demographic factors associated with emotional and social loneliness among older adults. [file 12877_2021_2058_MOESM1_ESM.docx]

# Supplementary material Socio-demographic characteristics associated with emotional and social loneliness among older adults

**Additional Table 1** Emotional and social loneliness sub scales of the 6-item De Jong-Gierveld Loneliness Scale.

| **Sub scale** | **Items** |
| --- | --- |
| Emotional loneliness | ‘I experience a general sense of emptiness’ |
|  | ‘I miss having people around’ |
|  | ‘I often feel rejected’ |
| Social loneliness | ‘There are many people I can trust completely’ |
|  | ‘There are plenty of people I can rely on when I have problems’ |
|  | ‘There are enough people I feel close to’ |

**Additional Table 2** Reliability tests (Cronbach’s α coefficients) of the 6-item De Jong-Gierveld Loneliness Scale and the 3-item emotional and social loneliness sub scales among 2251 participants of the UHCE study.

| Scale | **The United Kingdom**  n=548 | **Greece**  n=345 | **Croatia**  n=495 | **The Netherlands**  n=369 | **Spain**  n=494 |
| --- | --- | --- | --- | --- | --- |
| 6-item scale | .62 | .70 | .71 | .78 | .71 |
| 3-item sub scale emotional loneliness | .56 | .60 | .64 | .75 | .65 |
| 3-item sub scale social loneliness | .68 | .81 | .72 | .78 | .71 |

**Additional Table 3** Multivariable logistic regression models on associations between socio-demographic characteristics and emotional and social loneliness among 2251 participants of the UHCE study; using cut-off scores ≥1 and =3 for the sub scales of the De Jong-Gierveld Loneliness Scale.

|  | **Emotional loneliness** | | **Social loneliness** | |
| --- | --- | --- | --- | --- |
|  | Cut-off value ≥1  ‘yes’ n=1236 | Cut-off value=3  ‘yes’ n=256 | Cut-off value ≥1  ‘yes’ n=990 | Cut-off value=3  ‘yes’ n=323 |
|  | Multivariable model  OR (95% CI) | Multivariable model  OR (95% CI) | Multivariable model  OR (95% CI) | Multivariable model  OR (95% CI) |
|  |  |  |  |  |
| *Age (per 5 years)* | **1.20 (1.09-1.31)** | 1.12 (0.98-1.28) | 1.03 (0.94-1.13) | 1.00 (0.88-1.14) |
| *Sex (female)* | 1.17 (0.96-1.43) | **1.72 (1.23-2.41)** | 0.93 (0.76-1.14) | 0.82 (0.61-1.09) |
|  |  |  |  |  |
| *Living situation (without partner)* | **2.14 (1.75-2.61)** | **1.91 (1.39-2.62)** | 1.20 (0.98-1.46) | **1.45 (1.09-1.92)** |
| *Educational level*  Tertiary  Secondary  Primary or lower | ref. 1.28 (0.93-1.77) **1.77 (1.24-2.53)** | ref.  1.00 (0.58-1.73) 1.55 (0.88-2.75) | ref. 1.30 (0.93-1.81) 1.30 (0.91-1.86) | ref.  1.41 (0.83-2.38) **1.98 (1.12-3.52)** |
| *Migration background (yes)* | 1.15 (0.83-1.62) | 1.32 (0.84-2.09) | 0.90 (0.64-1.27) | 0.66 (0.42-1.04) |
|  |  |  |  |  |
| *Country*   The UK  Greece  Croatia  The Netherlands  Spain | ref.  **4.43 (3.11-6.31) 2.62 (2.01-3.41) 0.75 (0.56-0.99)** 1.15 (0.84-1.58) | ref.  **4.89 (2.77-8.61) 3.66 (2.32-5.75)** 1.70 (0.99-2.91) 1.63 (0.91-2.92) | ref.  **1.41 (1.00-1.99) 7.12 (5.40-9.39) 1.58 (1.19-2.10) 1.51 (1.10-2.08)** | ref. **2.30 (1.31-4.03) 8.65 (5.69-13.17)**  **2.58 (1.59-4.19)** 1.30 (0.74-2.29) |

Odds ratios and 95% confidence intervals are derived from multivariable logistic regression analyses for emotional and social loneliness (cut-off values Loneliness Scale ≥ 1 and =3). P-values <.05 in bold. OR=odds ratio; CI=confidence interval; ref.=reference group.

**Additional Table 4** Socio-demographic characteristics of 2251 participants of the UHCE study; by exclusive emotional and social loneliness and simultaneous emotional and social loneliness.

|  | **Total**  n=2251 | **Emotional loneliness exclusively (15.6%)**  n=351 | **Social loneliness exclusively (13.1%)**  n=294 | **Emotional and social loneliness simultaneously (13.6%)**  n=306 | **no loneliness (57.8%)** n=1300 | P-value |
| --- | --- | --- | --- | --- | --- | --- |
| Socio-demographic characteristics |  |  |  |  |  |  |
|  | Mean (SD)  n (% of total) | Mean (SD) n (%) | Mean (SD)  n (%) | Mean (SD)  n (%) | Mean (SD)  n (%) |  |
| *Age (in years)* | 79.7 (SD=5.6) | 79.8 (SD=6.1) | 80.3 (SD=5.0) | 80.7 (SD=5.4) | 79.3 (SD=5.7) | **<.001** |
| *Sex* |  |  |  |  |  | **<.001** |
| Female | 1360 (60.4%) | 244 (17.9%) | 156 (11.5%) | 219 (16.1%) | 741 (54.5%) |  |
| Male | 891 (39.6%) | 107 (12.0%) | 138 (15.5%) | 87 (9.8%) | 559 (62.7%) |  |
| *Living situation* |  |  |  |  |  | **<.001** |
| With partner | 1135 (50.4%) | 125 (11.0%) | 157 (13.8%) | 113 (10.0%) | 740 (65.2%) |  |
| Without partner | 1116 (49.6%) | 226 (20.3%) | 137 (12.3%) | 193 (17.3%) | 560 (50.2%) |  |
| *Educational level* |  |  |  |  |  | **<.001** |
| Tertiary | 206 (9.2%) | 28 (13.6%) | 26 (12.6%) | 15 (7.3%) | 137 (66.5%) |  |
| Secondary  Primary or less | 1426 (63.3%) 619 (27.5%) | 193 (13.5%) 130 (21.0%) | 206 (14.4%) 62 (10.0%) | 204 (14.3%) 87 (14.1%) | 823 (57.7%)  340 (54.9%) |  |
| *Migration background* |  |  |  |  |  | **.020** |
| No | 2066 (91.8%) | 325 (15.7%) | 259 (12.5%) | 274 (13.3%) | 1208 (58.5%) |  |
| Yes | 185 (8.2%) | 26 (14.1%) | 35 (18.9%) | 32 (17.3%) | 92 (49.7%) |  |
| *Country*  The UK | 548 (24.3%) | 74 (13.5%) | 48 (8.8%) | 27 (4.9%) | 399 (72.8%) | **<.001** |
| Greece | 345 (15.3%) | 97 (28.1%) | 28 (8.1%) | 49 (14.2%) | 171 (49.6%) |  |
| Croatia | 495 (22.0%) | 60 (12.1%) | 136 (27.5%) | 142 (28.7%) | 157 (31.7%) |  |
| The Netherlands | 369 (16.4%) | 51 (13.8%) | 35 (9.5%) | 44 (11.9%) | 239 (64.8%) |  |
| Spain | 494 (21.9%) | 69 (14.0%) | 47 (9.5%) | 44 (8.9%) | 334 (67.6%) |  |

P-values <.05 in bold. P-values for continuous variables were calculated with ANOVA and P-values for categorical variables were calculated with Chi-squared tests. SD=standard deviation.

**Additional Table 5** Multivariable logistic regression models on associations between socio-demographic characteristics and exclusive emotional loneliness; exclusive social loneliness; simultaneous emotional and social loneliness among participants of the UHCE study.

|  | **Emotional loneliness exclusively**  n=1652  ‘yes’ n=351 vs. ‘no emotional or social loneliness’ n=1300 | **Social loneliness exclusively** n=1603  ‘yes’ n=303 vs. ‘no emotional or social loneliness’ n=1300 | **Emotional and social loneliness simultaneously** n=1606  ‘yes’ n= 306 vs. ‘no emotional or social loneliness’ n=1300 |
| --- | --- | --- | --- |
|  | Multivariable model  OR (95% CI) | Multivariable model  OR (95% CI) | Multivariable model  OR (95% CI) |
| *Age (per 5 years)* | **1.15 (1.01-1.29)** | 1.12 (0.97-1.29) | **1.22 (1.06-1.40)** |
|  |  |  |  |
| *Sex (female)* | 1.22 (0.91-1.65) | **0.68 (0.50-0.93)** | 1.18 (0.85-1.64) |
|  |  |  |  |
| *Living situation (without partner)* | **2.55 (1.90-3.43)** | 1.06 (0.77-1.45) | **1.95 (1.43-2.67)** |
| *Educational level*  Tertiary  Secondary  Primary or lower | ref.  1.04 (0.65-1.67)  **1.68 (1.03-2.76)** | ref. 1.13 (0.69-1.84)  1.44 (0.82-2.51) | ref.  1.66 (0.90-3.06) **2.73 (1.42-5.24)** |
| *Migration background (yes)* | 0.98 (0.60-1.60) | 1.10 (0.69-1.75) | 0.98 (0.64-1.52) |
| *Country*   The UK  Greece  Croatia  The Netherlands  Spain | ref. **4.12 (2.62-6.47) 2.15 (1.44-3.21)** 1.14 (0.75-1.73)  1.03 (0.65-1.63) | ref.  1.42 (0.80-2.53)  **7.68 (5.22-11.28)**  1.20 (0.74-1.93)  1.16 (0.68-1.95) | ref. **5.65 (3.09-10.31)**  **13.80 (8.69-21.91)**  **2.67 (1.58-4.52)**  **1.83 (1.01-3.33)** |

Odds ratios and 95% confidence intervals are derived from multivariable logistic regression analyses for emotional and social loneliness (cut-off value Loneliness sub scales ≥ 2). P-values <.05 in bold. OR=odds ratio; CI=confidence interval; ref.=reference group.

**Additional Table 6** Participants in the UHCE study (n=2251) reporting emotional loneliness; by country and living situation.

| **Emotional loneliness** | | | | | | | | | | |
| --- | --- | --- | --- | --- | --- | --- | --- | --- | --- | --- |
|  | The United Kingdom  n=548 | | Greece n=345 | | Croatia n=495 | | The Netherlands n=369 | | Spain n=494 | |
|  | Lonely  n=101  n (%) | not lonely  n=447  n (%) | Lonely n=146  n (%) | not lonely n=199  n (%) | Lonely n=202  n (%) | not lonely n=293  n (%) | Lonely n=95  n (%) | not lonely n=274  n (%) | Lonely n=113  n (%) | not lonely n=381  n (%) |
| With partner  n=960 | 17 (8.0%) | 195 (92.0%) | 61 (34.9%) | 114 (65.1%) | 61 (40.7%) | 89 (59.3%) | 19 (10.2%) | 168 (89.8%) | 41 (17.4%) | 195 (82.6%) |
| With partner and children  n=175 | 2 (11.8%) | 15 (88.2%) | 20 (31.7%) | 43 (68.3%) | 14 (25.5%) | 41 (74.5%) | 0 (0.0%) | 3 (100.0%) | 3 (8.1%) | 34 (91.9%) |
| With children/ others  n=246 | 8 (19.5%) | 33 (80.5%) | 22 (59.5%) | 15 (40.5%) | 37 (42.0%) | 51 (58.0%) | 2 (40.0%) | 3 (60.0%) | 16 (21.3%) | 59 (78.7%) |
| Alone  n=870 | 74 (26.6%) | 204 (73.4%) | 43 (61.4%) | 27 (38.6%) | 90 (44.6%) | 112 (55.4%) | 74 (42.5%) | 100 (57.5%) | 53 (36.3%) | 93 (63.7%) |
